# Editorial: Oral Immune-Enhancing Research in Fish

**DOI:** 10.3389/fimmu.2022.850026

**Published:** 2022-03-17

**Authors:** Jorge Galindo-Villegas, Peter Bossier, Felipe E. Reyes-López

**Affiliations:** ^1^ Faculty of Biosciences and Aquaculture, Nord University, Bodø, Norway; ^2^ Laboratory of Aquaculture & Artemia Reference Center, Department of Animal Science and Aquatic Ecology, Faculty of Bioscience Engineering, Ghent University, Ghent, Belgium; ^3^ Centro de Biotecnología Acuícola, Facultad de Química y Biología, Universidad de Santiago de Chile, Santiago, Chile; ^4^ Facultad de Medicina Veterinaria y Agronomía, Universidad de Las Américas, Santiago, Chile; ^5^ Department of Cell Biology, Physiology and Immunology, Universitat Autònoma de Barcelona, Bellaterra, Chile

**Keywords:** teleost fish model, Omics, probiotics-immunobiotics, disease enhancement, endobolome, holistic analysis, selective manipulation, microbiome

## Introduction

Aquaculture is recognized globally as the fastest growing food-producing sector for human consumption (1). However, to satisfy increasing international demand, it is predicted that current production must be doubled by 2030 while improving the biosafety protocols (2). Such an expansion will necessarily involve implementing innovative actions in aquaculture production while also targeting effective health control measures and considering animal welfare.

Diet, environment, and host genotype have been repeatedly demonstrated to play an essential role in defining the fish gut composition (3). However, only a few studies have examined the association between disease severity and degree of dysbiosis in fish. Likewise, the management of pathogens and parasites remains a sustainability challenge industry-wide due to poor understanding of the associated fish immune mechanism across species, and the effects of climate change on aquaculture remain uncertain and complex to validate (4). While these concepts are not necessarily new, applying modern high-throughput sequencing approaches and state-of-the-art immune techniques will accelerate our understanding of the complex trophic (e.g., prokaryotic, eukaryotic) structures, the host recognition systems within aquaculture species and systems, and the effect of an intervention on eventual health outcomes allowing aquaculture to benefit from parallel advances in related disciplines (5).

Among the latest therapies developed, orally administered health promoters have demonstrated a strong ability as critical mediators in enhancing the fish immune system. However, the interaction between each element per fish species is highly complex and not always homogeneous along the different developmental stages. Therefore, to assess the expected potential immunological promotion and enhancement capacity, the use of dedicated, robust, and innovative molecular tools accompanied by keen strategies of analysis devoted to elucidating the mode of action for the tested compounds are required.

By collating evidence, eleven exciting research papers written by 79 authors in our Research Topic entitled “Oral Immune-Enhancing Research in Fish” are presented in this *ebook*. Together, they will provide a platform of discussion in the advancement of the actual knowledge and understanding beyond the immediate immune effects and the corresponding immune mechanisms in fish fed with functional feed additives, live supplemented feed, or even exposed to oral pollutants that stimulates a differential immune response in the animals under experimental or fish farm settings.

## Phytogenics as Sustainable Health Enhancers in Aquaculture Production

The implementation of innovative and sustainable production strategies has proven that plants have the potential to enhance the general physiology of animals. Phytogenics, defined as environmentally friendly plant-derived bioactive compounds, are used as functional feed additives that benefit animal growth and health. These elements often comprise aromatic plants extracts and essential oils rich in biologically active compounds. In farmed fish, a broad spectrum of phytogenics has been studied mainly due to their vast repertoire of properties, including growth promotion, antimicrobial, immunostimulant, antioxidant, anti-inflammatory, and sedative activities. Nevertheless, a pressing issue from using a phytogenic is the impact generated in the mucosal barrier and thus in the fish defense. Despite its enormous potential, establishing the phytogenics mode of action in mucosal tissues is essential for their proper use and safe administration Firmino et al..

Firmino et al. used feed microcapsules containing a blend of garlic essential oil, carvacrol, and thymol. They analyzed the Gilthead seabream skin transcriptional profile with some key skin mucus biomarkers and the resulting pathogenic bacterial growth capacity in the fish external live epithelium. Their results suggest that the phytogen-supplemented diet promoted the secretion of non-specific immune molecules into the skin mucus, causing the decrease of bacterial growth capacity. Likewise, novel transcriptomic enrichment analysis is presented, showing some cellular elements like the phagocytosis linked to the phytogenic treatment. Unfortunately, the exact mechanisms behind the action of the tested elements were not yet unveiled. Lastly, their results suggest that the tested additive could directly affect the intestinal host-microbial community composition that may influence the stress and immunological response in the skin. This observation suggests that the effect of the experimental diet on skin phagocytes and phagocytosis potential are a hot research line that deserves further evaluation.

Torrecillas et al. investigated the effect of two commercial additives in gill health of European sea bass fed with a low fish meal (FM)/low oil (FO) diet. The diets contained galactomannan oligosaccharides (Gmos) and a blend of garlic and labiatae-plant oils (Phyto), while an unsupplemented group served as a control. The morphological, histopathological, immunohistochemical, and biochemical parameters of the fish gills of sea bass given supplemented diets were protected from oxidative stress by modulating the expression patterns of oxidative enzyme-related genes. The diets also lowered the Pcna+ cell counts. It was argued that the reduction in Pcna counts in the gills of the fish-fed Phyto- and Gmos-supplemented diets might be associated with reduced levels of oxidative species via modulation of the endogenous antioxidant capacity. Moreover, Phyto supplementation reduced lamellar fusion, whereas Gmos supplementation reduced goblet cell count by 11% but did not affect the distribution of goblet cells along the primary lamellae. These protective effects could be considered in future prevention strategies against gill mucosal diseases in fish species, especially those sensitive to gill-associated pathogens.

In a different animal model, the gnotobiotic brine shrimp (*Artemia franciscana*), Zheng et al. showed that essential oils of *M. alternifolia* (containing mainly terpinen-4-ol and terpinene) and *L. citrata* (containing mainly citral and limonene) could significantly improve the survival of brine shrimp larvae when bath challenged with pathogenic *V. campbellii*. At the concentrations tested, no direct effect of the essential oils on the pathogen could be detected as revealed by life/dead staining and regrowth tests. The results rather indicated that supplementation of these two EOs enhanced immune gene expression, possibly contributing to protective immunity in brine shrimp larvae against *V. campbellii*. Furthermore, the EOs-regulate the expression of bacterial virulence factors, including decreased swimming motility and biofilm formation *in vitro*. This might contribute in part to protecting of the brine shrimp larvae against pathogenic *V. campbellii in vivo* However, further studies are needed to investigate the underlying protective mechanism of EOs, such as analyzing the expression of a larger panel of immune-related genes. Taken together, this study seems to indicate that essential oils can be part of disease intervention and/or prevention strategy in aquaculture.

## Selective Dietary Manipulation of the Microbiota Analyzed *In Vitro* and *In Vivo* Under Variated Contexts

Complex microbial communities colonize virtually every surface exposed to the external environment. They include prokaryotes, eukaryotes, and viruses, which provide enormous enzymatic capacity and play a key role in controlling many aspects of host physiology. Remarkably, the aquatic environment possesses an intrinsic aggressiveness due to the impressive number and diversity of microorganisms that coexist per square millimeter. Therefore, understanding the complex relationship between commensal microbes and fish is vital in maintaining healthy physiological homeostasis. Any disruption of this symbiosis (i.e., dysbiosis) can drive inflammation, and even worse, the development of inflammatory diseases (6). Indeed, studies using the 16s rRNA amplicon sequencing strategy have provided evidence that some pathologies are complex multifactorial diseases characterized by a profound dysbiosis of the microbial community. As a good example, in Atlantic salmon, it has been demonstrated that yellow mouth disease is dominated by distinct amplicon sequenced variants of *Tenacibaculum maritimum*, together with opportunistic taxa, including *Vibrio* spp., play a role in the clinical disease progression (7). Several strategies to generate a positive loop in maintaining host health through microbiota management have been proposed. Among the diverse strategies to selectively manipulate host-microbe interactions, diet is a significant factor that shapes the proportional representation of the microbiota present, particularly in the gut and their relative gene content (8). Reciprocally, the microbiota configuration influences the nutritional value of food Montalban-Arques et al. Although these approaches are particularly promissory, a critical issue determining a successful output from the potential promoters expected on providing solid immune indicators and showing a reliable disease enhancement at the time of dissecting responses is the robustness of the employed assays. In this special issue, we put forward solid pieces of research providing reliable evidence on the host-microbe relationships and the dietary manipulatory capacity.

The study performed by Firmino et al. presents an integrative analysis linking the gene transcriptional activity with the microbiota responses in the gut of gilthead seabream. The fish were fed with a regular diet supplemented with a functional feed containing microencapsulated essential oils from garlic, carvacrol, and thymol. The formula displayed an extensive holistic capacity of modulating the immune response and the microbial composition towards a beneficial profile characterized by the decrease in the classes *Bacteroidia* and *Clostridia*. Likewise, the feed additives increased the bacterial sequences associated with glutathione and lipid metabolism. Therefore, the study provides new insights into host-microbial metabolism’s shared role in building up a local immune response and crosstalk between gut and microbiota.

So far, the major challenge has been to understand the poorly studied signals and mechanisms of leukocytes after the addition of novel feed ingredients and their interaction with the microbiome at mucosal interphases in fish. Due to the unique characteristics displayed by the zebrafish model, exciting and solid progress in the recognition of new dietary elements and the associated mechanisms in the complex development and function of leukocytes and other types of cells with immune capacity has been documented (9). Here, Xie et al. using the powerful zebrafish as a model of intestinal inflammation show that excessive recruitment of leukocytes triggered by soybean meal can be reverted by applying a dietary sinomenine therapy. Sinomenine is an agonist of alpha7 nicotinic acetylcholine receptor (α7nAchR), a critical mediator controlling neuro-immune functions with anti-inflammatory properties through nicotine. Treated animals resulted with a higher proportion of CD4+ or Foxp3+ lymphocytes in the base of the intestinal folds, suggesting a synergy between α7nAchR and regulatory T-cells (Treg) in the response. Moreover, reduced enteritis and increased glucose metabolism were associated with the shift of *Sphingomonas* and *Shigella* composition and the increase in *Cetobacterium* biomass, suggesting that sinomenine can prevent fish foodborne enteritis at the immune or microbiota levels.

To gain further insights on the impact that dietary modulators have over the host commensal microorganisms and immunity, Castejón et al. investigated the integral role of the microbiota upon immunity and antibody processing in response to an extended endocrine disruptor in the aquatic environment in the Gilthead seabream. Specifically, the 17α-ethinylestradiol (EE_2_) and the agonist G protein-coupled estrogen receptor agonist G-1 (G1) in the gut of gilthead seabream. Humoral lytic and oxidative stress response, essential mucosal inflammatory marker genes, and goblet cells contribution to immunity at the gut mucosal surface were explored. Additionally, the microbiome landscape on the immune repertoire following the dietary treatment and a priming immunization protocol using a model antigen (KLH) was revealed. Minor changes were observed on the stress markers or the gene expressions. However, the dietary treatment with EE2 combined with the immunization promoted remarkable changes in the bacterial class Betaproteobacteria and, unexpectedly, on the estrogen-degrading genus *Novosphingobium* a major member of the endobolome. Overall, this study highlighted the intrinsic relationship between xenoestrogens and their associated receptors in the ubiquitous fish immune regulation. Moreover, the subtle but significant crosstalk with the fish gut endobolome was described in fish for the first time.

There is an increasing consensus of the relevance for evaluating *in vitro* the biological mode of action of isolated compounds before moving forward to *in vivo* trials Firmino et al.. From the immunomodulatory point of view, amino acids (AA) are crucial components in the biosynthesis of immune support-associated molecules. In fish, several articles reported the effect of the dietary administration of AA on a spectrum of immune-related functions (10), with no particular attention on the cell-specific modulatory mechanisms. Thus, this necessitates *in vitro* studies that provide fast lights about the response dynamic for specific immune cell subsets prior to the *in vivo* stage. Machado et al. showed that methionine and tryptophan perform distinct roles in the immune response of head kidney leukocytes (HKL). While L-methionine increased the modulation of the expression of pro-inflammatory gene markers, tryptophan augmented the expression of the anti-inflammatory counterparts. However, the increase was only perceived when the HKL were exposed to lipopolysaccharides-extracted or UV-inactivated *Photobacterium damselae* subsp. *pisicida (Phdp)*. Exposing HKL to *Phdp* produces and releases an exotoxin that induces apoptosis. Therefore, the exposure of HKL to the exotoxin resulted in a diminished signal of apoptosis in the L-methionine- incubated cells. Meanwhile, the L-tryptophan group showed a pro-apoptotic activity by the elevated expression of the initiator *caspase 8* and an increased activity of caspase 3, suggesting a lowered resilience in the L-tryptophan-treated HKL in response to the infection by *Phdp*.

## Classic Probiotic Bacteria, Immunostimulants, and Novel Recombinant Proteins

Probiotics and immunostimulants have been used as preventive strategies for limiting and controlling fish diseases. Probiotics supplementation is aimed to maximize the profit from the crosstalk between the host and the commensal microbiota for improving the fish robustness and resilience. The study of Muñoz et al. used the *Lactococcus lactis* – a commensal bacteria found in mammals and salmonids, for producing the recombinant type Ia interferon (rIFN-Ia). Likewise, its role in protecting against the infectious pancreatic necrosis virus (IPNV) following *in vitro* and *in vivo* strategies was assessed. The study showed *in vitro* upregulation of antiviral genes (*mx*; *pkr*) after the administration of different doses of rIFN-IA and a reduction of the IPNV viral load determined through qPCR. The oral administration of Atlantic salmon (*Salmo salar*) with the *L. lactis* rIFN-Ia, revealed a higher expression of antiviral-related genes in spleen rather than head kidney. In addition, it showed a lower IPNV load in live Atlantic salmon even up to 60 days post-infection, suggesting that this oral strategy improved the antiviral response. Adopting a similar strategy, Santibañez et al. evaluated the effect of the *L. lactis* recombinant rIFN-γ-producer oral administration on rainbow trout (*Oncorhynchus mykiss*) through a challenge against 10^8^ CFU of *F. psychrophilum*. The higher survival rate on rainbow trout fed *L. lactis* rIFN-γ suggests that this application would be a useful novel strategy for conferring oral protection to fish against the infection of some extracellular bacterial pathogens. However, further evaluations of the system *in vivo* and using variated developmental stages are required.

On the other hand, yeasts are considered immune modulators since they contain β-glucan, mannans, chitin, and nucleic acids, that can be recognized as microbial-associated molecular patterns (MAMPs) by pattern recognition receptors (PRRs) with high capacity of training the innate memory in fish myelocytes Zhang et al. The study of Morales-Lange et al. used the *Cyberlindnera jadinii* yeast in a 40% soybean meal (SBM) diet. The perspective of the study is interesting because they propose the use of the spleen to evaluate the effect of the novel diet fed in Atlantic salmon (*Salmo salar*). The study includes the evaluation of the immune response by RNAseq and indirect ELISA. Furthermore, the *C. jadinii* was exposed to two different down-stream processing conditions: heat-inactivated (ICJ), or autolysation (ACJ). In line with previous effects reported in the intestine after the dietary administration of SBM, these authors confirmed the pro-inflammatory status in spleen, being characterized by the increased TNFα and IFNγ levels recorded. Notably, ACJ response was characterized by the increasing level of IL-10 and the diminished content of TNFα. hus, the authors suggest using ACJ as a component in the formulation of diets for sustainable Atlantic salmon farming.

β-glucans have been extensively used in the aquaculture industry. However, the species-specificity, length of their side chains, and frequency of administration have depicted a variable role in immunomodulation, showing differences between short- and long-treatments, including the modulation of stress-associated mechanisms and immunosuppression. Cornet et al. proposed the use of Gas1a β-glucan produced by the null-mutant yeasts Gas1 of *Saccharomyces cerevisiae*, as new immunostimulant to protect rainbow trout (*Oncorhynchus mykiss*) juveniles against *Aeromonas salmonicida achromogenes* infection. For comparison purposes, the study included three β-glucan preparations: wild-type (WT), MacroGard, and the novel Gas1, evaluated at two time points after feeding the animals. Unexpectedly, after 36 days, the dietary supplementation with MacroGard resulted in the downregulation of immune genes and the lysozyme activity, immunoglobulin production and neutrophils proliferation in comparison to Gas1. The resulting immune status produced by Gas1 correlated with a higher survival rate against *A. salmonicida*. Moreover, the survival rate difference between Gas1 and WT suggests lower degree of branching and shorter side chain length may be the responsible in the differences observed in the efficiency of the immuno-stimulation between both β-glucan preparations.

Overall, the contributions gathered in this Research Topic provide holistic evidence on various mechanisms associated with oral immune enhancement of fish at the cellular and immune level, propose methods on the selective manipulation of the gut microbiota, and more importantly, deal on how to provide an increased health status and disease resistance in cultured teleost fish. By putting this information together, we hope to contribute to improving fish aquaculture by providing new concepts and research trends. Notably, we intend to provide evidence that by utilizing robust methodologies accompanied with robust analytical strategies, functional, exciting novel findings and future research perspectives could undoubtedly be achieved.

## Author Contributions

All authors listed have made a substantial, direct, and intellectual contribution to the work, and approved it for publication.

## Conflict of Interest

The authors declare that the research was conducted without any commercial or financial relationships that could be construed as a potential conflict of interest.

## Publisher’s Note

All claims expressed in this article are solely those of the authors and do not necessarily represent those of their affiliated organizations, or those of the publisher, the editors and the reviewers. Any product that may be evaluated in this article, or claim that may be made by its manufacturer, is not guaranteed or endorsed by the publisher.
